# Diet Quality Assessment and the Relationship between Diet Quality and Cardiovascular Disease Risk

**DOI:** 10.3390/nu13124305

**Published:** 2021-11-28

**Authors:** Kristina S. Petersen, Penny M. Kris-Etherton

**Affiliations:** 1 Department of Nutritional Sciences, Texas Tech University, Lubbock, TX 79409, USA; 2 Department of Nutritional Sciences, Pennsylvania State University, University Park, State College, PA 16802, USA; pmk3@psu.edu

**Keywords:** diet quality, dietary patterns, cardiovascular disease, Healthy Eating Index, Alternate Healthy Eating Index, Mediterranean Diet Score, Healthful Plant-Based Diet Index, Dietary Approaches to Stop Hypertension Score

## Abstract

Cardiovascular diseases (CVD) are the leading cause of morbidity and mortality in the U.S. and globally. Dietary risk factors contribute to over half of all CVD deaths and CVD-related disability. The aim of this narrative review is to describe methods used to assess diet quality and the current state of evidence on the relationship between diet quality and risk of CVD. The findings of the review will be discussed in the context of current population intake patterns and dietary recommendations. Several methods are used to calculate diet quality: (1) a priori indices based on dietary recommendations; (2) a priori indices based on foods or dietary patterns associated with risk of chronic disease; (3) exploratory data-driven methods. Substantial evidence from prospective cohort studies shows that higher diet quality, regardless of the a priori index used, is associated with a 14–29% lower risk of CVD and 0.5–2.2 years greater CVD-free survival time. Limited evidence is available from randomized controlled trials, although evidence shows healthy dietary patterns improve risk factors for CVD and lower CVD risk. Current dietary guidance for general health and CVD prevention and management focuses on following a healthy dietary pattern throughout the lifespan. High diet quality is a unifying component of all dietary recommendations and should be the focus of national food policies and health promotion.

## 1. Introduction

Cardiovascular diseases (CVD), primarily ischemic heart disease (IHD) and stroke, are the leading causes of morbidity and mortality in the U.S. and globally [1]. Between 1990 and 2019, the number of CVD cases globally approximately doubled from 257 to 285 million to 523 million. Cardiovascular deaths have also increased in the U.S., most notably from 2010 to 2018 [2]. Of the modifiable CVD risk factors (i.e., metabolic, environmental, and behavioral) contributing to CVD burden, dietary risks are the number one ranked behavioral risk factor. In 2017, it was estimated that dietary risk factors contributed to 53% of CVD deaths and 58% of CVD-related disability [3].

Dietary risk factors are defined by intake patterns that are suboptimal, which includes overconsumption dietary components associated with increased risk of disease or low intakes of dietary components associated with reduced risk of disease. Diet quality is a term used to quantify the overall healthfulness of a dietary pattern based on its constituents. Assessment of diet quality aligns with the current understanding that the totality of the diet has a greater effect on health outcomes than individual dietary components including nutrients, foods, and bioactives [4,5]. The aim of this narrative review is to briefly describe common methods used to assess diet quality and summarize the current state of evidence on the relationship between diet quality and risk of CVD. We will describe the findings of our review in the context of current population intake patterns and dietary recommendations for general health and CVD prevention and management.

## 2. Common Methods Used to Define Diet Quality

Assessment of diet quality provides a summary of the overall pattern of intake of a population that can be linked to risk of diet-related diseases including CVD. Several methods are used to calculate diet quality that can be broadly classified as follows: (1) a priori indices based on dietary recommendations; (2) a priori indices based on foods or dietary patterns associated with risk of chronic disease; (3) exploratory data-driven methods. In the following sections, these methods of diet quality assessment will be briefly described.

### 2.1. Indices Based on Dietary Recommendations

Diet quality indices based on adherence to national dietary recommendations have been developed in some countries. For example, the Healthy Eating Index (HEI) was developed in 1995 to assess adherence to the Dietary Guidelines for Americans [6]. The original HEI was updated in 2005 (HEI-2005) [7]; this was followed by the release of updated iterations in 2010 (HEI-2010) [8] and 2015 (HEI-2015) [9] to align with the release of updated editions of the Dietary Guidelines for Americans. The maximum attainable score for the HEI is 100 (Table 1); a higher score represents greater adherence to the Dietary Guidelines for Americans and higher diet quality. The components included in the HEI are based on the Dietary Guidelines for Americans recommendations; scoring criteria as well as detailed methodology for HEI calculation and statistical software code are published by the National Cancer Institute [10].

Since 2005, each iteration of the HEI has been evaluated for content validity, construct validity, and reliability [18,19,20]. The evaluation of the HEI-2015 demonstrated that use of the index on an exemplary menu yielded high scores (87.8 to 100 points) [20]. In addition, sufficient variation in scores among individuals was shown when the HEI-2015 was applied to NHANES 2011–12 data (range 32.6 to 81.2 points), and differentiation was achieved by the HEI-2015 when it was applied to groups with known differences in diet quality. The HEI-2015 provides a measure of diet quality independent of total energy intake, which supports construct validity. Reliability (internal consistency) was also demonstrated in this evaluation [20]. Finally, when the HEI-2015 was applied to data from the NIH-AARP prospective cohort study, the highest diet quality (quintile 5) was associated with lower risk of all-cause (men: HR 0.80, 95% CI 0.78–0.82; women: HR 0.77, 95% CI 0.74–0.80), cancer (men: HR 0.78, 95% CI 0.74–0.82; women: HR 0.80, 95% CI 0.75–0.86), and CVD mortality (men: HR 0.87, 95% CI 0.83–0.92; women HR 0.79, 95% CI 0.73–0.85) compared to the lowest diet quality (quintile 1) supporting the use of the HEI-2015 to predict health outcomes and criterion validity [20].

### 2.2. Indices Based on Foods or Dietary Patterns Associated with Risk of Chronic Disease

Most diet quality indices are developed based on epidemiological research that shows an association between intake of foods or dietary patterns and chronic disease risk. Many of these types of diet quality indices have been developed [21]; here we describe the most commonly used indices (Table 1).

The Alternate Healthy Eating Index (AHEI) was developed to assess intake of foods and macronutrient sources associated with reduced chronic disease risk based on prior observational research [15]. The AHEI was updated in 2010 (AHEI-2010) to include additional dietary factors involved in the development of chronic disease [16]. Higher diet quality measured by both the AHEI and AHEI-2010 is associated with lower risk of chronic disease [15,16]. In an analysis of data from the Women’s Health Initiative Observational Study, a cohort of postmenopausal women, those with an AHEI Score in quintile 5 had a 23% lower risk of CVD (HR 0.77, 95% CI 0.70–0.84) and a 30% lower risk of heart failure (HR 0.70, 95% CI 0.59–0.82) compared to quintile 1 [22]. In a prospective analysis of U.S. male health professionals, the highest AHEI quintile had a 20% (RR 0.80, 95% CI 0.71–0.91) reduction in risk of major chronic disease (CVD, cancer, or death) as well as a 39% (RR 0.61, 95% CI 0.49–0.75) reduction in CVD risk compared to quintile 1 [15]. Similar risk reductions for major chronic disease (RR 0.89, 95% CI 0.82–0.96) and CVD (RR 0.72, 95% CI 0.60–0.86) were observed for U.S. female health professionals with high AHEI Scores [15]. In another analysis of the Nurses’ Health Study and Health Professionals Follow-up Study, participants in the highest quintile for the AHEI-2010 Score had significantly lower risk of major chronic disease (RR 0.81, 95% CI 0.77–0.85), CVD (RR 0.76, 95% CI 0.71–0.81), CHD (RR 0.69, 95% CI 0.62–0.76), stroke (RR 0.80, 95% CI 0.71–0.91), diabetes (RR 0.67, 95% 0.61–0.74), and cancer (RR 0.94, 95% CI 0.89–0.98) after 24 years of follow-up compared to those in the lowest quintile [16].

Several indices have been developed based on the Mediterranean diet, which is a traditional dietary pattern consumed by countries in the Mediterranean region associated with lower risk of chronic disease. The original Mediterranean Diet Score (MDS) was developed by Trichopoulou et al. and included eight components characteristic of a Greek Mediterranean diet (high monounsaturated fat to saturated fat ratio, moderate intake of alcohol, high intake of legumes, cereals (including bread and potatoes), fruits, vegetables, and low intake of meat and meat products and milk and dairy products) [17]. Additional components were added to the original MDS to create a ten-point MDS [11]. Trichopoulou et al. reported that a two-point increase in the ten-point MDS was associated with a significant reduction in the risk of all-cause (HR 0.75, 95% CI 0.64–0.87), CHD (HR 0.67, 95% CI 0.47–0.94), and cancer (HR 0.76, 95% CI 0.59–0.98) mortality in a Greek cohort [11]. The ten-point MDS was subsequently adapted to develop the Alternate Mediterranean Diet Score (AMED), which contains nine components: vegetables (excluding potatoes), fruits, nuts, whole grains, legumes, fish, ratio of monounsaturated to saturated fat, red and processed meats, and alcohol [12]. In the Nurses’ Health Study, the highest AMED quintile had lower risk of CHD (RR 0.71, 95% CI 0.62–0.82), fatal CHD (RR 0.58, 95% CI 0.45–0.75), and non-fatal CHD (RR 0.78, 95% CI 0.66–0.93) compared to the lowest quintile. Similar reductions in the risk of CVD (HR 0.77, 95% CI 0.65–0.93), CHD (HR 0.69, 95% CI 0.53–0.91), and stroke (HR 0.67, 0.0.47–0.96) were observed in a prospective analysis of women with type 2 diabetes participating in the Women’s Health Initiative when the highest diet quality assessed by the AMED (quintile 5) was compared with the lowest diet quality (quintile 1) [23]. A number of other Mediterranean-diet-based indices have been developed that are used in research [24]. For example, the Mediterranean Diet Adherence Screener (MEDAS), a 14-point index based on the Spanish Mediterranean diet, was used in the PREDIMED study [25].

More recently, the Healthful Plant-Based Diet Index (HPDI) was developed based on epidemiological links between higher intake of healthy plant-based foods and lower risk of chronic disease [13]. The HPDI positively weights healthful plant foods and negatively weights animal foods and unhealthy plant-based foods. Analyses of the Nurses’ Health Study and Health Professionals Follow-up Study have shown that per 10 unit increase in the HPDI, the risk of CHD (HR 0.88, 95% CI 0.85–0.91) [26] and type 2 diabetes (HR 0.83, 95% CI 0.8–-0.85) [13] is reduced.

The Dietary Approaches to Stop Hypertension (DASH) Score is also commonly used to define diet quality. The DASH Score is based on the DASH diet that significantly reduced blood pressure in a randomized controlled-feeding study [27]. Many versions of the DASH Score are used by researchers; however, the DASH Score developed by Fung et al. [14] is the most widely used [28]. Fung et al. [14] define the DASH Score according to eight food and nutrient components: fruits, vegetables, whole grains, nuts and legumes, low-fat dairy, red and processed meats, sweetened beverages, and sodium. In an analysis of data from the Nurses’ Health Study, the highest adherence to the DASH Score was associated with lower risk of total CHD (RR 0.73, 95% CI 0.64–0.84), fatal CHD (RR 0.66, 95% CI 0.52–0.83), non-fatal CHD (RR 0.78, 95% CI 0.66–0.91), and total stroke (RR 0.83, 95% CI 0.71–0.96) compared to lowest adherence [14].

### 2.3. Data-Driven Methods for Assessing Diet Quality

A posteriori approaches are used to identify population-specific dietary patterns. These approaches rely on statistical methods such as factor analysis, including principal component analysis and common factor analysis, or cluster analysis [5]. Principal component analysis is a variable-reducing procedure that creates linear combinations (components, factors, or patterns) based on the correlation or covariance matrices of the original variables. These principal components are then correlated with health outcomes to assess the relationship with dietary patterns of differing diet quality. Similarly, factor analysis identifies common underlying dimensions (factors or patterns) of intake and aggregates food items or food groups based on the correlation strength [5].

Cluster analysis aggregates individuals within a dataset into relatively homogeneous subgroups (clusters) based on the frequency of foods/foods groups consumed, absolute or standardized food/food group or nutrient intake, or percentage of calories from food sources/food groups or nutrients [5]. These clusters can then be used to explore associations between patterns of intake and health outcomes. In contrast to a priori diet quality indices, a posteriori approaches are treated as exploratory and hypothesis-generating since the findings are specific to the dataset used for the analyses. The focus of this review is on epidemiological research examining a priori diet quality indices, since this is of greater relevance than a posteriori approaches to disease prevention and management.

## 3. Epidemiological Evidence: Diet Quality and CVD Risk

Consistently, epidemiological evidence shows higher diet quality is associated with lower risk of CVD [28,29]. In the sections that follow, prospective cohort studies examining the relationship between diet quality and the relative and absolute risks of CVD in adults will be summarized. In addition, studies investigating the association between changes in diet quality and CVD risk will be described.

### 3.1. Diet Quality and Relative Risk of CVD

Numerous prospective cohort studies have shown that higher diet quality, assessed by various indices, is associated with lower relative risk of CVD events or mortality [28,29,30]. In a 2020 systematic review and meta-analysis of prospective cohort studies, higher diet quality (highest category) assessed by the HEI (13 studies; relative risk (RR) 0.80, 95% CI 0.77–0.84), the AHEI (21 studies; RR 0.77, 95% CI 0.74–0.80) and the DASH Score (31 studies; RR 0.81, 95% CI 0.78–0.85) was associated with a 19–23% relative risk reduction in CVD incidence or mortality compared to the lowest diet quality category [29]. In a pooled analysis, including 31 studies that measured diet quality by the HEI, AHEI, or DASH indices, the highest diet quality was associated with a 20% reduction in the relative risk (RR 0.80, 95% CI 0.78–0.82) of incident CVD or CVD mortality.

Recently, Shan et al. conducted comprehensive analyses examining the relationship between diet quality, as assessed by four different indices, and CVD risk in initially healthy women (Nurses’ Health Study I and II) and men (Health Professionals Follow-up Study) followed for up to 32 years. The authors reported that the highest diet quality quintile for the HEI-2015, AMED, HPDI, and AHEI had a 14–21% lower risk of CVD compared to quintile 1 [31]. Of note, the median diet quality scores for quintile 5 (highest diet quality) in these analyses are well below the maximum score possible for the HEI-2015 (75–78 points out of 100), AMED (34–35 points out of 45), HPDI (64 points out of 90), and AHEI (59–61 points out of 100), although the scores are substantially higher than those achieved by quintile 1 (HEI-2015: 52–55 points, AMED: 20 points, HPDI: 46–47 points, AHEI: 33–35 points). This indicates that higher diet quality scores, even at below-ideal values, confer CVD benefit. In further support, in this study, each 25-percentile increase in the HEI-2015 (multivariate adjusted HR 0.80, 95% CI 0.77–0.83), AMED (HR 0.90, 95% CI, 0.87–0.92), HPDI (HR 0.86, 95% CI 0.82–0.89), and AHEI (HR 0.81 95% CI, 0.78–0.84) was associated with a significant reduction in CVD risk. Several subgroup analyses were also conducted and, consistently, the association between higher diet quality and lower CVD risk was observed, although, in some subgroups, the magnitude of the association differed. Thus, this study demonstrates that greater adherence to healthy dietary patterns, defined by high diet quality, is associated with lower risk of CVD, and similar risk reductions are observed across various indices of diet quality.

### 3.2. Diet Quality and Absolute Risks for CVD across the Lifespan

Commonly, epidemiological analyses of diet quality and CVD risk present relative risk estimates, which are a comparison of the percentage of individuals who develop CVD when exposed to higher diet quality vs. the percentage of individuals who develop CVD when exposed to lower diet quality. Alternatively, absolute risk estimates compare CVD risk in one group vs. another group (i.e., higher diet quality vs. lower diet quality) and are more applicable to clinical practice and patient outcomes.

A recent prospective cohort study demonstrated that higher diet quality is associated with markedly lower absolute risk of incident CVD. In this analysis that included data from six prospective cohort studies, higher baseline diet quality was associated with lower absolute 30–40 year risks of incident CVD in young (20–39 years), middle-aged (40–59 years), and older (60–79 years) adults [32]. For young men (Q1 14.3% vs. Q5 5.9%) and women (Q1 8.8% vs. Q5 3.0), there was a two- to three-fold difference in 40-year risk of incident CVD with the highest diet quality vs. lowest diet quality measured by the AHEI-2010. In middle-aged men (−8.9 percentage points) and women (−13.4 percentage points) and older men (−5.0 percentage points) and women (−5.8 percentage points), those in the highest vs. lowest quintile for the AHEI-2010 had substantially lower 30-to-40-year risk of incident CVD. Similar absolute risk reductions were observed for the DASH and the AMED indices. Notably, higher diet quality (highest quintile) measured by the AHEI-2010 was associated with an additional 0.5–2.2 years of CVD-free survival compared to quintile 1 [32]. These analyses show higher diet quality is associated with significant reductions in absolute CVD risk, as well as longer CVD-free survival time. This was also demonstrated in a previous analysis where higher diet quality measured by the AHEI-2010 was associated with greater life expectancy (up to 5 years, depending on sex and age) in a U.S. cohort of health professionals [33].

### 3.3. Diet Quality Changes and CVD Risk

Strong and consistent epidemiologic evidence shows higher diet quality is associated with lower relative and absolute risk of CVD as well as greater CVD-free survival time. Further support of the CVD benefits of higher diet quality comes from studies that have evaluated the relationship between changes in diet quality over time and CVD risk. In a prospective analysis of U.S. health professionals, a 20-percentile increase in diet quality, over a 12-year period, measured by the AHEI (HR 0.85, 95% CI 0.76–0.96) and AMED (HR 0.93, 95% CI 0.88–0.99), was associated with significantly reduced risk of death from CVD [34]. In another set of analyses using the same cohort of U.S. health professionals, those with the greatest 4-year improvement in diet quality assessed by the AHEI (HR 0.92, 95% CI 0.87–0.99), AMED (HR 0.93, 95% CI 0.85–1.02), and DASH Score (HR 0.93, 95% CI 0.87–0.99) had lower risk of CVD than those whose diet quality remained relatively stable [35]. Furthermore, an increase in diet quality, assessed by the AHEI (HR 0.93, 95% CI 0.88–0.99) and AMED (HR 0.91, 95% CI 0.86–0.97), during the first four years of follow-up was associated with lower CVD risk during the next 20 years. Thus, improvements in diet quality over time are associated with lower risk of CVD regardless of the diet quality index used.

## 4. Clinical Trial Evidence: Diet Quality and CVD Risk

Randomized controlled trials provide the highest level of evidence for establishing causal relationships between dietary exposures and health outcomes. However, it is inherently difficult to conduct long-term randomized controlled trials to examine the effect of dietary exposures on health outcomes, especially hard endpoints such as CVD [36]. Few randomized controlled trials have been conducted to examine the effect of improved diet quality on CVD incidence or mortality. While there are many randomized controlled trials examining the effect of dietary interventions on CVD risk factors, typically the effect of the dietary intervention on diet quality or changes in diet quality are not reported. Therefore, this limits inferences about the effect of dietary interventions on diet quality and the effect of improving diet quality on CVD risk.

The landmark PREDIMED study, a three-arm parallel randomized primary prevention trial, showed that a Mediterranean diet with extra virgin olive oil (HR 0.69, 95% CI 0.53–0.91) or nuts (HR 0.72, 95% CI 0.54–0.95) reduced the risk of a composite CVD outcome (acute myocardial infarction, stroke, or death from cardiovascular causes) after a median of 4.8 years follow-up compared to a control group given education to follow a low-fat diet [25]. In this trial, diet quality was measured by the 14-point Mediterranean diet adherence score that showed the two Mediterranean diet groups had higher diet quality over the follow-up period. The difference in diet quality between the Mediterranean diet groups and the control group over years 1 to 6 of follow-up was 1.4 to 1.8 points, a relatively modest increase in diet quality [25]. Thus, PREDIMED demonstrated that small changes in diet quality confer clinically relevant CVD risk reductions.

While there is a paucity of clinical trial data on the effect of improving diet quality, assessed by a priori indices, on CVD incidence or risk factors for CVD, substantial evidence shows that healthy dietary patterns such as the Mediterranean diet [37], the DASH diet [27,38], the Portfolio Dietary Pattern [39], and the dietary patterns recommended by the Dietary Guidelines for Americans [40] improve risk factors for CVD. Based on the available evidence, the 2020 Dietary Guidelines Advisory Committee concluded that there is strong and consistent evidence that dietary patterns associated with decreased risk of CVD are characterized by higher intake of vegetables, fruits, whole grains, low-fat dairy, and seafood and lower intake of red and processed meat, refined grains, and sugar-sweetened foods and beverages [4]. Thus, current dietary guidance for general health and CVD risk reduction is focused on achieving a high-quality healthy dietary pattern [41,42,43,44].

## 5. Current Population Level Diet Quality

Diet quality is suboptimal both globally and in the U.S. [41,45]. At a global level, in 2019, the Summary Exposure Value (SEV) for dietary risks was 47, which indicates that 47% of the world’s population has poor diet quality [45]. In this analysis, diet quality was defined by suboptimal intake of fruits, vegetables, whole grains, seafood, nuts and seeds, fiber, legumes, polyunsaturated fatty acids, and milk coupled with the overconsumption of sodium, trans fats, sugar sweetened beverages, and processed meats.

Based on the most recent data for the U.S. (NHANES 2015–2016), the mean HEI-2015 Score for Americans ≥2 years of age is 59 out of 100 [41]. Of particular concern is the decline in diet quality that is observed across childhood. In 2015–2016, children aged 2–4 years had a mean HEI-2015 Score of 61 points with progressive declines in the score from ages 5–8 years (mean HEI-2015 Score 55 points), 9–13 years (52 points), and 14–18 years (51 points) [41]. In adulthood, the mean HEI-2015 Score for the 19–30 years group was 56 with slightly higher scores observed for the 31–59 age group (59 points) and the 60+ age group (63 points).

It is of concern that there has been little change in HEI-2015 Scores over time. In 2005–2006, the mean HEI-2015 Score for Americans ≥2 years of age was 56 points, and over the decade through 2015–2016, diet quality increased by 3–4 points [41]. Thus, at a U.S. population level, adherence to the Dietary Guidelines is poor, which is reflected by low HEI Scores. This highlights the critical importance of population-wide policies to increase diet quality to reduce the burden of preventable diet-related chronic diseases including CVD.

## 6. Dietary Recommendations for CVD Prevention and Management

All of the recommended dietary patterns for general health and CVD prevention and management emphasize high diet quality. Much attention is given to the differences between dietary guidance issued by different authoritative organizations, and the concordance amongst dietary recommendations is often not the topic of public discourse, leading to public confusion and skepticism of dietary recommendations. In Table 2, we have summarized dietary recommendations for general health and CVD prevention and management (Dietary Guidelines for Americans 2020–2025, American Heart Association/ American College of Cardiology, and the National Lipid Association), which highlights the considerable overlap and consistency between the guidelines [41,42,43,44]. Broadly, the guidelines consistently recommend high intakes of vegetables, fruits, wholegrains, legumes, nuts and seeds, low-fat dairy, unprocessed lean meats and poultry, and seafood, and lower intakes of sources of saturated fat, added sugar, and sodium. Differences are observed for saturated fat recommendations based on the purpose of the guidelines (i.e., general health <10% kcal, management of lipids/lipoproteins (<7%), or CVD prevention/management (5–6% kcal); however, consistently, it is recommended that saturated fat intake is limited, and sources of unsaturated fats are consumed instead [41,42,43,44]. Intake of a high-quality diet is therefore a unifying component of all dietary recommendations. To promote general health and reduce the burden of chronic diseases such as CVD, attention should be focused on improving diet quality by increasing consumption of core-healthful foods and lowering consumption of energy-dense, nutrient-poor foods.

## 7. Conclusions

Higher diet quality is defined by greater adherence to recommended dietary patterns and/or intake of dietary patterns associated with lower risk of CVD and other chronic diseases. Substantial evidence from prospective cohort studies demonstrates that higher diet quality, regardless of the index used, is associated with lower risk of CVD and greater CVD-free survival time across adulthood. Limited evidence is available from randomized controlled trials, although the available evidence shows healthy dietary patterns improve risk factors for CVD and lower CVD risk. Thus, current dietary guidance for general health and CVD prevention and management focuses on following a healthy dietary pattern throughout the lifespan. While there are modest differences in the specific food- and nutrient-based recommendations issued by authoritative organizations, high diet quality is a defining feature of all recommended dietary patterns for general health and CVD prevention and treatment. There is consensus that a healthy diet is abundant in vegetables, fruits, wholegrains, legumes, nuts and seeds, low-fat dairy, and lean protein foods, and is low in saturated fat, added sugar, and sodium. Thus, rather than debating recommendations for specific foods, food groups, or nutrients, attention should be focused on the totality of the diet and areas of relative agreement about core-healthful foods and developing strategies that can effectively shift the population towards higher diet quality. Dietary change is challenging and requires intervention at the government (policy), community, sociocultural, and individual levels. However, the available evidence suggests that strategies to improve the diet quality at the population level will have substantial health benefits and be cost-effective [46,47,48]. High diet quality is a unifying component of all dietary recommendations and should be the focus of efforts to promote general and cardiovascular health.

## Figures and Tables

**Table 1:** Components and scoring criteria of commonly used diet quality indices [9,11,12,13,14,15,16,17].

Components	Diet Quality Indices							
	HEI-2015 [9] 0–100 Points	AHEI [15] 0–87.5 Points	AHEI-2010 [16] 0–110 Points	Original MDS [17] 0–8 Points	MDS [11] 0–9 Points	AMED [12] 0–9 Points	HPDI [13] 0–90 Points	DASH [14] 0–8 Points
Positively Weighted Components	Total fruits (5 points; ≥0.8 c-eq/1000 kcal)			Fruits and nuts (1 point; ≥median)	Fruits and nuts (1 point; ≥median)	Total fruits (1 point; ≥median)		Total fruits (1 point; 4.1 servings/day)
	Whole fruits (5 points; ≥0.4 c-eq/1000 kcal)	Whole fruits (10 points; ≥4 servings/day)	Whole fruits (10 points; ≥4 servings/day)				Whole fruits (5 points; >highest quintile)	
	Total vegetables (5 points; ≥1.1 c-eq/1000 kcal)	Vegetables excluding potatoes (10 points; ≥5 servings/day)	Vegetables excluding potatoes (10 points; ≥5 servings/day)	Vegetables (1 point; ≥median intake)	Vegetables (1 point; ≥median intake)	Vegetables excluding potatoes (1 point; ≥median intake)	Vegetables excluding potatoes (5 points; >highest quintile)	Vegetables excluding potatoes and legumes (1 point; 4.6 servings/day)
	Greens and beans (5 points; ≥0.2 c-eq/1000 kcal)							
	Whole grains (10 points; ≥1.5 c-eq/1000 kcal)	Cereal fiber (10 points; 15 g/day)	Wholegrains (10 points; men ≥90 g/day, women ≥ 75 g/day)	Cereals including bread and potatoes (1 point; ≥median intake)	Cereals including bread and potatoes (1 point; ≥median intake)	Whole grains (1 point; ≥median intake)	Whole grains (5 points; >highest quintile)	Wholegrains (1 point; 2.4 servings/day)
	Dairy (10 points; ≥1.3 c-eq/1000 kcal)							Low-fat dairy (1 point; 2.3 servings/day)
	Total protein foods (5 points; ≥2.5 c-eq/1000 kcal)	White meat: red meat ratio (10 points; 4)						
	Seafood and plant proteins (5 points; ≥0.8 c-eq/1000 kcal)	Nuts and soy protein (10 points; ≥1 serving/day)	Nuts and legumes (10 points; ≥1 serving/day)	Legumes (1 point; ≥median intake)	Legumes (1 point; ≥median intake) Fish (1 point; ≥median intake)	Legumes (1 point; ≥median intake) Nuts (1 point; ≥median intake) Fish (1 point; ≥median intake)	Legumes (5 points; >highest quintile) Nuts (5 points; >highest quintile)	Nuts and legumes (1 point; 1.5 servings/day)
	Fatty acids (10 points; (PUFA + MUFA)/SFA ≥2.5)	PUFA:SFA ratio (10 points; ≥1)	Long chain n-3 fats—EPA and DHA (10 points; 250 mg/day) PUFA (10 points; ≥10% energy)	MUFA: SFA ratio (1 point; ≥median intake)	MUFA: SFA ratio (1 point; ≥median intake)	MUFA: SFA ratio (1 point; ≥median intake)	Vegetables oils (5 points; >highest quintile)	
		Alcohol (7.5 points; men 1.5–2.5 servings/day, women 0.5–1.5 servings/day)	Alcohol (10 points; men 0.5–2.0 servings/day, women 0.5–1.5 servings/day)	Ethanol (1 point; moderate undefined)	Ethanol (1 point; men 10–50 g/day, women 5–25 g/day)	Ethanol (1 point; 5–25 g/day)		
		Multivitamin use (7.5 points; ≥5 years)						
							Tea and coffee (5 points; >highest quintile)	
Negatively Weighted Components	Refined grains (10 points; ≤1.8 oz. eq/1000 kcal)						Refined grains (5 points; <lowest quintile)	
	Sodium (10 points; ≤1.1 g /1000 kcal)		Sodium (10 points; lowest decile)					Sodium (1 point; <1041 mg/day)
	Added sugars (10 points; ≤6.5% energy)		SSB and fruit juice (10 points; 0 servings/day)				Fruit juice (5 points; < lowest quintile) SSB (5 points; <lowest quintile) Sweets and desserts (5 points; <lowest quintile)	SSB (1 point; 0 servings/day)
	Saturated fat (10 points; ≤8% energy)		Red and processed meat (10 points; 0 servings/day)	Meat and meat products (1 point; <median intake) Dairy products (1 point; <median intake)	Meat and poultry (1 point; <median intake) Dairy products (1 point; <median intake)	Red and processed meat (1 point; <median intake)	Animal fat (5 points; <lowest quintile) Meat (5 points; <lowest quintile) Eggs (5 points; <lowest quintile) Other animal-based foods (5 points; <lowest quintile) Fish and seafood (5 points; <lowest quintile) Dairy (5 points; <lowest quintile)	Red and processed meat (1 points; <0.4 servings/day)
		Trans fat (10 points; ≤0.5% energy)	Trans fat (10 points; ≤0.5% energy)					
							Potatoes (5 points; <lowest quintile)	

AHEI: Alternate Healthy Eating Index; AHEI-2010: Alternate Healthy Eating Index-2010; AMED: Alternate Mediterranean Diet Score; c-eq: cup equivalent; DASH: Dietary Approaches to Stop Hypertension; HEI-2015: Healthy Eating Index-2015; HPDI: Healthful Plant-Based Diet Index; MDS: Mediterranean Diet Score; oz-eq: ounce equivalent.

**Table 2:** Dietary recommendations for general health and CVD prevention and management.

Recommendations	Dietary Guidelines for Americans 2020–2025 [41]			American Heart Association/American College of Cardiology [42]	National Lipid Association [43]
	Daily Amount of Food from Each Group (2000 Kcal)				Nutrition Recommendations for Management of Dyslipidemia
	Healthy U.S.-Style Dietary Pattern	Healthy Mediterranean-Style Dietary Pattern	Healthy Vegetarian Dietary Pattern	AHA Eating Pattern Based on The Healthy U.S.-Style Eating Pattern	
Vegetables (cup eq/day)	2 ½	2 ½	2 ½	2 ½	Follow a healthy dietary pattern, including an emphasis on a variety of plant foods and lean sources of protein e.g., DASH, USDA (healthy U.S.-style), AHA, Mediterranean style, and vegetarian/vegan
Dark-green vegetables (cup eq/week)	1 ½	1 ½	1 ½	1 ½	
Red and orange vegetables (cup eq/week)	5 ½	5 ½	5 ½	5 ½	
Beans, peas, lentils (cup eq/week	1 ½	1 ½	1 ½	1 ½	
Starchy vegetables (cup eq/week)	5	5	5	5	
Other vegetables (cup eq/week)	4	4	4	5	
Fruit (cup eq/day)	2	2 ½	2	2	
Grains (oz. Eq/day)	6	6	6 ½	6	
Whole grains (oz. Eq/day)	≥3	3	3 ½	3	
Refined grains (oz. Eq/day)	<3	3	3	3	
Dairy (cup eq/day)	3	2	3	3	
Protein foods (oz. Eq/day)	5 ½	6 ½	3 ½	5 ½	
Meats, poultry, eggs (oz. Eq/week)	26	26	3 (eggs)	26	
Seafood (oz. Eq/week)	8	15	6	8 (preferably oily)	
Nuts, seeds, soy products (oz. Eq/week)	5	5	8 Soy Products 7 Nuts and Seeds	5	
Beans, peas, lentils (cup eq/week)			6		
Oils (g/day)	27	27	27	45	
Limit on calories for other uses (kcal/day)	240	240	250		
Limit on calories for other uses (% kcal/day)	12	12	13		
Saturated fat (% kcal/day)	<10			6 (solid fats)	<7
Added sugars (% kcal/day)	<10			5	<10 (avoid if triglycerides >500 mg/dayl)
Sodium (mg/day)	<2300			1787	<2300
Dietary cholesterol (mg/day)	No recommendation (diets have <300 mg/day)				<200
Fiber (g/day)	28			31	5–10 (viscous)
Plant sterols and stanols (g/day)					~2

## Data Availability

Not applicable.
